# Positive Psychological Impacts of Cooking During the COVID-19 Lockdown Period: A Qualitative Study

**DOI:** 10.3389/fpsyg.2021.635957

**Published:** 2021-03-18

**Authors:** Ozan Güler, Murat İsmet Haseki

**Affiliations:** ^1^Department of Gastronomy and Culinary Arts, Faculty of Tourism, Mersin University, Mersin, Turkey; ^2^Department of Business Administration, Kozan Faculty of Business Administration, Çukurova University, Adana, Turkey

**Keywords:** COVID-19, psychological impact, hedonic well-being, eudaimonic well-being, leisure characteristics, cooking

## Abstract

This study aims to explore the positive psychological effects of culinary experiences during the COVID-19 lockdown days. Qualitative research methods adopted to provide a deeper understanding. Data was collected through a structured online survey from 30 participants in Turkey. This occurred between April 10th and June 3rd, 2020 when the strict confinement measures were applied. Content analysis was deductively applied according to the Stebbins’s Theory of Casual vs. Serious Leisure which classifies the well-being according to characteristics of leisure experiences. The results revealed that at the first stage people went into the kitchen with the motivation of pure happiness and relaxation indicating hedonic well-being. However, people who intended to spend time with culinary activities with the expectations of pure happiness left the kitchen with eudaimonic outcomes by gaining special skills and knowledge, self-actualization and self-enrichment. When these outcomes are evaluated based on the Stebbins’s theoretical framework, culinary activities have both casual and serious leisure experience characteristics in terms of psychological well-being. It is understood that culinary activities have versatile leisure characteristics. Thanks to the culinary activities, people do not only obtain pure happiness and relaxation but can draw wider inferences about their life by realizing their own potential during the psychologically challenging COVID-19 lockdown days.

## Introduction

The 21st century has already been faced with several outbreaks of viral diseases [e.g., Severe Acute Respiratory Syndrome (SARS-CoV), Middle East Respiratory Syndrome (MERS-CoV), and Ebola Hemorrhagic Fever (EHF)]; however, none of them have resulted in a collective war to save the entire human civilization. The last one emerged in late December of 2019 (Ahmed et al., 2020). A case of unknown cause was detected in Wuhan, China, which first reported to the WHO Country Office in China on December 31, 2019 (Ahmed et al., 2020; Gössling et al., 2020; Rodríguez-Rey et al., 2020). In a short time, the cases kept on rising and later confirmed to be affected by a new type of coronavirus called COVID-19 (SARS-CoV-2) by the World Health Organization (Meng et al., 2020). By mid-March, global air travel had already transported the virus from Wuhan to all continents and was formed in 146 countries (Ahmed et al., 2020; Gössling et al., 2020). On March 11, 2020, the WHO upgraded the status of the COVID-19 outbreak which turned from an epidemic to a pandemic (Ahmed et al., 2020; Gössling et al., 2020; Rodríguez-Rey et al., 2020). The infection rate accelerated through community transmission and by the end of April confirmed cases reached approximately two million in over 200 countries. From December 31, 2019 to November 9, 2021, reported cases of COVID-19 exceeded 103 million including 2,236,453 deaths [European Centre for Disease Prevention and Control (ECDC), 2021]. Turkey, which is the sample of this research, is one of the countries to be most severely affected by the COVID-19 pandemic. Turkey is the 9th ranking in terms of the number of total cases and 18th ranking in terms of the number of total deaths among the 221 countries. As of February 08, 2021, reported cases of COVID-19 exceeded 2.5 million including 26,900 deaths and over 63,000 active cases in Turkey (Worldometers, 2021). Therefore, this unprecedented epidemic has become the top priority problem to be tackled by Turkey as well. The lack of an effective vaccine to prevent the disease and/or limited medical interventions to treat it caused countries to respond to the pandemic with some non-pharmaceutical interventions. (NPIs) including lockdown, home isolation, voluntary/required/mandatory quarantine, social distancing including vulnerable or whole populations, closing of almost all locations such as schools/universities and non-essential businesses/workplaces. This caused the canceling or postponing of events and bans on gatherings of people over specific numbers (Gössling et al., 2020; Kaplan et al., 2020). In 2020 as well as many countries in the world, Turkey applied to the partial and full-time lockdown measures involving the large part of the society between the dates of April 11, 2020 and June 3, 2020.

While this unexpected disease has caused people from all over the world to become infected with the virus and even die, it has also started to create psychological pressure on them (Rodríguez-Rey et al., 2020). It is quite understandable that such a sudden outbreak causes severe damages to the mental health of both the infected people and those who are in a close contact with them (Ahmed et al., 2020). Previous epidemiological studies about the effect of SARS and MERS epidemics revealed that the survivors have suffered depression, anxiety, negative psychological effects, panic attack, psychomotor excitement, psychotic symptoms, delirium and even suicidal tendency (Ahmed et al., 2020; Rodríguez-Rey et al., 2020). These findings are remarkably close to the COVID-19 pandemic as well. Studies on the psychological effects of COVID-19 have been conducted in many countries. These countries include China, Italy, and Spain, where the pandemic is present with the highest numbers. Of those reported some negative psychological effects ranging from moderate to severe levels such as stress, fear, anxiety, emotional reactions, depression, insomnia, vulnerability to excessive harmful alcohol use, inadequate supplies, inadequate information, financial loss, and other associated with psychological problems (Ahmed et al., 2020; Bodrud-Doza et al., 2020; Brooks et al., 2020; Cerami et al., 2020; Duan and Zhu, 2020; Meng et al., 2020; Ozamiz-Etxebarria et al., 2020; Rodríguez-Rey et al., 2020; Qiu et al., 2020; Parlapani et al., 2020; Rossi et al., 2020; Wang et al., 2020). Some of the studies conducted on people living in China explored the prevalence and risk factors of acute posttraumatic stress symptoms in participants (Liu et al., 2020; Sun et al., 2020). As a result, the anxiety and worry increasing during the process has started to turn the emotion felt as psychological pressure into psychological trauma. These mental and psychological difficulties experienced by people have visibly led to a severe psychological disorder on everyone, regardless of children, youngsters, adults, and elders. COVID-19 has resulted in self-isolation and physical distancing for millions of people (Giles and Oncescu, 2020).

The unexpected isolation due to coronavirus disease showed that leisure is important, and people can still find ways to be together (Lashua et al., 2020; Samdahl, 2020). People who had to stay at home have started to spend time with a series of daily leisure activities to keep their psychology healthy and to deal with their fear and feelings of restriction of freedom (Bond et al., 2020; Cheval et al., 2020; Easterbrook-Smith, 2020; Gammon and Ramshaw, 2020; Giles and Oncescu, 2020; Rodríguez-Rey et al., 2020; Son et al., 2020; Su et al., 2020). According to the research conducted by Rodríguez-Rey et al. (2020) in the Spanish sample, talking to someone on the phone, instant messaging or video calls, browsing or sharing social network contents, watching films or shows, watching TV, reading and practicing sports or physical exercise (48,7%) were the most frequent leisure activities. Su et al. (2020), in their study comparing Wuhan and Lombardy samples, found that the stress of individuals living in Lombardy decreased as they increased their domestic leisure recreation. Based on increasing leisure time and decreasing leisure activity opportunities, Bond et al. (2020) stated that people have been turning to generating makeshift leisure opportunities and made the “Toilet Roll Challenge” activity, which has been a trend in Twitter, their research topic. At this point, culinary activities have begun to be seen as an opportunity for many people to get away from a problem they have never encountered before. Since shopping resources are restricted, dining out is limited to only take away, people over and under a certain age are completely forbidden to go out, and hygiene is the top priority. People have started to do many things that they bought before at home. Gammon and Ramshaw (2020) stated that in the period of enforced lockdowns and social distancing, people resort to nostalgia-based leisure activities such as baking bread and making fresh pasta. Easterbrook-Smith (2020) affected by the “bake your own bread” trend on social media, prepared a publication on how bread-baking became an efficient leisure recreation activity when the coronavirus cases were getting worse. The return of these activities is classified as providing sustenance, filling newly available leisure time and offering a way to demonstrate one’s skill and activities on social media.

The culinary activities, which were considered as a necessity at the beginning of COVID-19 lockdown days in the world, turned into a leisure/recreational activity over time and began to function as a tool in improving people’s psychological well-being. Photos of dishes shared on social media and often tried for the first time to illustrate this fact in particular. From this point of view, the positive effects of in-door culinary activities on the psychology of people during the mandatory COVID-19 quarantine days were investigated within the scope of well-being and leisure experience characteristics concepts. As a result, the purpose of this study is mainly twofold; (i) explore the positive psychological impacts (hedonic/eudaimonic) of culinary activities during the COVID-19 lockdown days and (ii) to discover the leisure experience characteristics (casual/serious) of culinary activities.

## Literature Review

### Well-Being and Stebbins’s Theory of Casual Versus Serious Leisure

Current research on psychological well-being based on two philosophical approaches to the good life. On one hand, there is the “hedonic” approach, which expresses good life as the pursuit of “pure happiness.” On the other hand, there is the “eudaimonic” approach that explains good life with concepts such as meaning, personal growth, identity building (Voigt et al., 2010). Hedonic well-being, also considered as subjective well-being, is generally measured in three dimensions as “frequent positive feeling,” “infrequent negative feeling” and “life satisfaction” (Diener, 1984, 1994). On the other hand, eudaimonic well-being claims that reaching pure happiness is possible with psychological emotions that will help people reach their real capacity and make life more meaningful, rather than short-term emotions such as pleasure (Ryff, 1989). Eudaimonic well-being is mostly measured by variables such as “purpose in life,” “personal growth,” “self-acceptance,” “environmental mastery,” “autonomy” and “positive relations with others” (Ryff and Keyes, 1995; Ryff and Singer, 2008). Waterman (2005) argued that hedonic well-being will not be sufficient for the effects people perceive from activities, and that the eudaimonic perspective with outcomes such as self-determination, balance of challenge and skills, effort, and self-realization values should be considered. According to Waterman (2005), hedonic and eudaimonic well-being outputs together constitute “hedonic enjoyment” and the eudaimonic contribution of an activity alone does not make sense. Ryan and Deci’s (2001) conceptualization could be more helpful to clarify the discussion of two-dimensional well-being (see Table 1.).

**TABLE 1:** Hedonic and eudaimonic well-being.

Hedonic well-being	Eudaimonic well-being
(Subjective well-being)	(Psychological well-being)
• Presence of positive mood	• Sense of control or autonomy
• Absence of negative mood	• Feeling of meaning and purpose
• Satisfaction with various domains of life	• Feeling of belongingness
• Global life satisfaction	• Social contribution
	• Competence
	• Personal expressiveness
	• Personal growth
	• Self-acceptance

*Source: Ryan and Deci (2001).*

Whereas psychology in the second half of the 20th century was mainly about repairing damage and curing diseases, we nowadays find a considerable number of studies on human flourishing and developing positive qualities (Meyers et al., 2013). Few researchers explored the concepts of hedonic and eudaimonia at the activity level (Voigt et al., 2010), however, leisure and recreational experiences are considered as a valuable research topic as one of the domains that create common well-being (Uysal et al., 2016). Previous research has indicated that participation to the leisure activities is positively linked to both physical health and psychological wellbeing (Caldwell, 2005). There has been a number of precious researches done on the importance of leisure activity as a prerequisite for a healthy and happy life (Shin and You, 2013). The vast majority of the studies show that leisure vacation experiences and activities have a significant effect on both tourists’ (Hunter-Jones, 2003; Gilbert and Abdullah, 2004; Pesonen and Komppula, 2010; Voigt et al., 2010; Kim et al., 2015) and residents’ (Boukas and Ziakas, 2016; Rivera et al., 2016; Suess et al., 2018) life satisfaction and wellbeing (Uysal et al., 2016). Studies revealed that these significant and positive effects come into prominences in a variety of life domains such as family life, social life, leisure life, cultural life, among others (Uysal et al., 2016).

When the subject of well-being is investigated based on the leisure activities, Stebbins’s “theory of casual vs. serious leisure” is one of the very first theoretical concepts that comes to mind. This theory provides a conceptual framework for leisure types based on the hedonic and eudaimonic well-being outputs. Serious leisure and casual leisure have served as two contrasting concepts for describing leisure experiences. Stebbins (1997a,b) states that the hedonic outcomes of an activity indicate casual characteristics. Accordingly, active or passive leisure activities that provide casual experience include short-term and momentary happiness, pleasure, relaxation, socialization and sensory stimulations in terms of the well-being outputs (Stebbins, 1997a, 2008). Contrary to casual leisure, serious leisure activities may provide long-term and enduring psychological benefits such as obtaining special knowledge or skills, self-actualism, self-enrichment, feeling of accomplishment, belongingness to a special world, identity building and career development (Stebbins, 1997a,b, 2008). Although Stebbins never associated the serious leisure with the concept of eudaimonic well-being directly, the characteristic of the serious leisure is in the way he describes it is quite similar with the outcomes of eudaimonic well-being of Voigt et al. (2010). This theoretical framework put forward by Stebbins has formed the basis for research in the following years. In particular, the concept of serious leisure has been the major focus of the following studies. When the studies are examined it has been shown that some research focused on the development of serious leisure scales (Gould et al., 2008; Munusturlar-Akyıldız and Argan, 2016), some studies took the elder people into the sample within the context of hobby and sports competitions (Brown et al., 2008; Heo et al., 2012, 2013; Cheng et al., 2017) and some others delved into exploring serious characteristics of sports competitions, adventure events, dance events, and sport tourism (Jones, 2000; Gibson et al., 2002; Kane and Zink, 2004; Green and Jones, 2005; Brown, 2007; Dilley and Scraton, 2010).

### Culinary Activities as a Tool for Well-Being

It is not possible for every leisure or recreational activity to produce useful outcomes for positive psychological well-being (Caldwell and Smith, 2006). However, “anecdotal evidence suggests that the act of cooking encourages creativity, engages the senses, provides a way to nurture others, and is immediately gratifying” (Black, 2009). Some clinicians believe that cooking can help to relieve depression by promoting positive activity, increasing goal-oriented actions, and motivating clients to take an active role in their rehabilitation (Whalen, 2014). The literature mostly benefited from the evidence-based cooking interventions to promote change in various areas of physical health, such as weight, BMI, blood pressure, diabetes prevention, or increased vegetable consumption (Reicks et al., 2014, 2018). Cooking activities have been discussed in the psychology and health literature with the aim of investigating their positive effects on the psychological, psychosocial and physical wellness of the disabled, sick, elderly, and disadvantaged individuals, mostly through cooking interventions (Marquis et al., 2001; Fitzsimmons and Buettner, 2003; Hill et al., 2007; Huang et al., 2009; Bier et al., 2011; Lock et al., 2012; Jyväkorpi et al., 2014; Barak-Nahum et al., 2016; Foloppe et al., 2018).

There have been quite limited efforts to handle recreative culinary activities based on its’ psychological effect on well-being through psychological theories (Mosko and Delach, 2020). In the literature, there is limited number of studies conducted for the psychological effects of cooking in ordinary times without any intervention (Daniel et al., 2011; Mosko and Delach, 2020) and in the COVID-19 pandemic period (Easterbrook-Smith, 2020; Gammon and Ramshaw, 2020). While Daniel et al. (2011) examined therapeutic benefits of cooking through the eyes of amateur kitchen chefs, regardless of well-being, Mosko and Delach (2020), in their research they used qualitative and quantitative methods together; they examined the effects of university students’ cooking behaviors on their well-beings. As a result, both studies have demonstrated that cooking has the potential to offer much more than meeting physiological needs, most particularly at the point of improving mood and emotions, promoting social and familial connections, and enhancing the individual creativity and awareness. Gammon and Ramshaw’s (2020) research in the context of “baking bread” and “making fresh pasta” and Easterbrook-Smith’s (2020) in the context of “baking bread” put forward some positive psychological effects such as coping with negative emotions, relieving from boredom with an enjoyable leisure activity and offering a way to demonstrate one’s skill and activities.

## Materials and Methods

### Study Design

This study adopted a qualitative research approach in order to get a deeper understanding of the psychological effect of cooking activities during the COVID-19 lockdown days. This descriptive and exploratory aim of the study required qualitative research design. When it comes to understanding a person’s life, story or behavior, qualitative research becomes a valuable technique. Qualitative methods can be used to uncover and understand what lies behind any phenomenon about which little is yet known. Besides, it can be used to gain new and fresh knowledge about an issue which is lesser known (Strauss and Corbin, 1990: 19). As a result, the main reasons for preferring qualitative research design were both the direct aim of the research to learn about the individual experiences of the people and collecting the answers in a compelling period (under home lockdown) that had never been experienced before.

### Sample

Based on the purpose of the research, the heterogeneous purposive sampling method (maximum variation sampling) (Saunders, 2012) was used. To provide the maximum variation during the data collection, heterogeneity was emphasized in terms of criteria such as marital status, gender, profession, age, educational status and place of residence. The first condition to provide this heterogeneous sample is that the participants should have turned to cooking at home during the lockdown period. Therefore, those who are necessarily engaged in cooking (e.g., housewives and cooks) and those who do not enter the kitchen volunteer excluded from the study sample. In order to include the right people in the sample, (i) people personally known by the researchers based on their social media posts, (ii) people the researchers knew by asking their surroundings and (iii) snowballing from previous contacts were consulted. As it is known, a sample size of 12–30 people for qualitative research is thought to be sufficient to explain the phenomenon of a research (Creswell, 2007; Lee J. Y. et al., 2010; Saunders, 2012). The sample size was determined as 30, which is the upper limit. Before being interviewed, participants were individually checked *via* telephone whether they met the eligibility criteria or not. As a criterion for inclusion in the study, the questions “Have you prepared any meals or drinks that you tried to make for the first time during the coronavirus pandemic days? Can you tell them with their names and stories?” questions were asked to the participants. Those who answered “No” to this question were not included in the study. When the demographic characteristics of the individuals participating in the study are examined, it is known that 20 of the 30 participants are female, 17 are single, and 18 of them have undergraduate and graduate education. The youngest participant is 20 years old while the oldest one is 53. The overall average age of the whole is 30,9. The participants live in 14 different cities in Turkey.

### Data Collection and Procedure

A structured qualitative online survey consisting of closed and open-ended questions was used as a data collection tool. Interviews can be realized with respondents on a one-to-one basis by an exchange of e-mails (Rodham and Gavin, 2006). In qualitative research, the traditional method is based on collecting data by asking questions prepared before they were face to face and under record (Neville et al., 2016). However, in some cases, it may not be possible to reach the sample face to face. This may be due to some situational reasons or the vulnerable nature of the sample (Henrickson, 2007). For those respondents who need greater reassurance of confidentiality and privacy, or who feel threatened by the electronic presence of others in focus groups. This form of interview may offer an acceptable alternative. The online interview approach also offers greater space for in-depth investigation of the perspectives of the respondents, thereby allowing the nuanced feelings and attitudes inherent in an emotionally charged subject to be explored (Rodham and Gavin, 2006). The two mandatory practices of the COVID-19 period, such as lockdown and social distancing, have made the online survey technique almost indispensable, as many researchers use. In Turkey, the curfew announced for the elders (65+) on March 21, 2020, enforced on specific days and times. This includes all segments of society until June 6, 2020. For this reason, the data in this study were collected with the internet-based approach and online interview form. The access link of the interview form was sent to the e-mail addresses of the people who met the criteria for participation in the study. Before answering the questions, the participants were informed about the purpose of the research and they were reminded that participation in the study was voluntary.

The structured online surveys were conducted over “Google Forms” between April 10th and June 3, 2020. People from different cities of Turkey participated in the interviews. It took approximately 15–30 min for the participants to answer the questions. The Online survey consisted of two parts. While there are six demographic questions in the first part, there are four open-ended questions in the second part. Participants were asked not to give short answers as “yes” or “no” to the open-ended questions, and it was stated that they could answer the questions in any length they want. The online interview form included the following four open-ended questions; (1) How many hours on average did you spend a day in the kitchen during the quarantine period? If you think you have spent more time than in the past, could you please share with us the factors that motivate you to do so? and (2) What kind of benefits do you think spending time in the kitchen during the quarantine period brings you? All the answers obtained from these four questions were used in the research findings. As it can be understood from the questions, the participants were not directly asked about the psychological effects of their culinary experiences and technical expressions that could manipulate them were avoided.

### Data Analysis

All qualitative methods have a common purpose in that they aim to explain a complex phenomenon from the viewpoint of those who encounter it (Vaismoradi et al., 2013). For researchers who prefer to use a relatively low level of interpretation, the use of qualitative descriptive methods such as descriptive phenomenology, content analysis, and thematic analysis are acceptable compared to grounded theory or hermeneutic phenomenology, where a higher level of interpretive complexity is needed (Vaismoradi et al., 2013). It is a systematic coding and categorizing technique used to explore vast volumes of textual information in a discreet manner in order to determine trends and patterns of terms used, their frequency, relationships, and contact structures and discourses (Mayring, 2000). Content analysis may be sufficient for simple reporting of common issues found in the data. In the data analysis process, a five-step process that consists of “description and interpretation,” “modalities of approaches,” “consideration of context of data,” “data analysis process,” and “evaluation of the analysis” was operated (Vaismoradi et al., 2013). The 30 electronically recorded interviews were transcribed verbatim by the first researcher and then subjected to the qualitative content analysis. Within the scope of the purpose of the research, the deductive approach was used in determining the themes (Voigt et al., 2010). A deductive approach is useful when the aim of content analysis is to test a previous theory or to compare categories in a different situation (Hsieh and Shannon, 2005; Elo and Kyngäs, 2008). As mentioned before the theoretical background of the study is based on “Stebbins’s Theory of Casual vs. Serious Leisure Experience.” The existing main themes are “hedonic/casual leisure experience” and “eudaimonic/serious leisure experience.” Hedonic/casual leisure experience involved the pleasure, relaxation, and sensory stimulation whereas eudaimonic/serious leisure experience included special knowledge or skills, fulfilling and identity-building, belongingness to a special world, career development and effort/perseverance (Voigt et al., 2010). Firstly, participants’ answers to unstructured questions were interpreted and converted into codes. Only the frequency of codes is not applied due to danger of missing the context (Morgan, 1993). In addition, based on the general meaning of the comments of the participants, inferences were made regarding the theme of the comments. These codes were then placed under sub-themes that they belonged to. In the last stage, sub-themes are placed under the hedonic/casual and eudaimonic/serious main themes to which they belong. The researchers involved in the study made the interpretation of the codes in regard to themes separately. The resulting disputes and uncertainties were sent to two separate field experts who were not involved in the study. The evaluation of the analysis process was then done.

## Results

### Motivational Effects of Cooking on Well-Being

Findings obtained as a result of online interviews with 30 participants were presented in two tables. The first question is “How many hours on average did you spend a day in the kitchen during the lockdown period? Could you please share the factors motivating you to do so with us?” Based on this, in the context of well-being, the expectations of the participants from spending time in the kitchen during the COVID-19 period were understood. According to the answers, it was understood that the average time the participants spend in the kitchen is 3.5 h. The least amount of time spent in the kitchen stated as 2 h, while the one spending the most time stated about 8 h. The most common time was 3 h. All the participants stated that these times they spend in the kitchen are longer than before the lockdown. Table 2 reports the factors that motivate participants to engage in cooking during the lockdown period. Since some participants stated more than one motivation factor, the total number of statements is over 30, which is the total number of participants. When 51 codes obtained from 30 participants are placed in the relevant themes, casual/hedonic characteristics of well-being in the kitchen are dominant, as it is clearly seen in Table 2. Among the answers given to the question “What are the factors motivating you to spend time in the kitchen?” no item in the serious/eudaimonic characteristics category was encountered. Therefore, it can be said that the basic psychological expectation that directs individuals to spend time in the kitchen during the COVID-19 quarantine days is hedonic well-being and, from Stebbins’ point of view, engaging in culinary activities is initially perceived as a casual leisure.

**TABLE 2:** Hedonic/casual vs. eudaimonic/serious characteristics of cooking motivations (*n* = 30).

Casual/hedonic characteristic (Σ_*i* =_ 51)	Σ_*i*_
**Pleasure (Σ_*i* =_ 27)**	
Relief from boredom	13
Spending time with a quality event	5
Spending pleasant time with loved ones	4
Gain admiration/appreciation	4
Physical Activity	1
**Sensory stimulation (Σ_*i* =_ 24)**	
Desire to taste new flavors	17
Desire to prepare clean and hygienic meals	5
Constant desire to eat	2

*Since some participants stated more than one motivation factor, the total number of statements is above the total number of participants.*

From the Table 2, it is seen that the major factor of casual/hedonic motivation is having “Pleasure and Fun.” Based on the 25 statements, people motivated to engage in culinary activities as a kind of tool for relieving their boredom, spending time with a quality event, spending pleasant time with the people living with them, gaining their appreciation with what they prepare and doing physical activities. These motivations, which are included in the “active entertainment” group in Stebbins’ approach, can be supported by the verbatim quotes from the different participants.

*“I spend at least 2–3 h a day in the kitchen. I want to relieve my boredom and cooking makes me happy” (Participant 7)*.

*“The main reason I spend more time in the kitchen is my son. Having my son with us during this period and his desire to taste different flavors is the biggest share. Being with him in the kitchen is the biggest motivation for me” (Participant 1)*.

*“Spending time in the kitchen with my daughter becomes a supportive program along with her activities at other times of the day. While we motivate her by doing culinary activities during the day, we are all motivated by her laughing” (Participant 29)*.

*“It has been a means of finding peace for me. Trying new recipes has become a way to forget the bad thoughts in my mind” (Participant 26)*.

*“We do many activities at home. But it is certain that we spend the best and most productive time in the kitchen” (Participant 22)*.

Apart from the “Pleasure and Fun” expectations of the participants, the other dimension that creates casual/hedonic motivations is “Sensory Stimulation.” Expressions under this factor are related to eating, and the main motivation has been “cooking novel foods.” For people, cooking dishes, appetizers and desserts that they have never tried to cook at home or have never tasted before but wondered about their taste were in the top priority to be in the kitchen. In addition, people who do not want to eat outside or refrain from shopping due to the COVID-19 pandemic stated that they spend time in the kitchen due to their cleanliness and hygiene expectations. Lastly, two participants stated that the source of their motivation to spend time in the kitchen is to have a “constant desire to eat”:

*“We are in the period of fasting right now. Since I don’t normally have enough time to be in the kitchen to make new products, I wanted to prepare some new and different menus. I used to prefer to prepare fast and easy meals before the quarantine days” (Participant 8)*.

*“The fact that we cook everything ourselves and pay more attention to hygiene in this period naturally increased the time spent in the kitchen. What leads us to this is our avoidance of all kinds of ready-to-eat food in terms of hygiene and attention to cleanliness” (Participant)*.

“The feeling of being locked in the house increased my desire to eat something during the quarantine. Therefore, timelessly, I found myself preparing something in the kitchen” (Participant 28).

### Psychological Effects of Cooking on Well-Being

The second question asked to the participants in the context of the purpose of the study was “What kind of benefits do you think spending time in the kitchen during the lockdown brings you?” Based on this, it was aimed to classify the positive psychological effects perceived by the participants in the context of “well-being.” Table 2 shows the effects of culinary activities perceived by the participants as hedonic/casual versus eudaimonic/serious well-being. Just like the situation examined in terms of motivation, the total number of statements is over 30, which is the total number of participants, since some participant stated more than one positive psychological return. When 86 codes obtained from 30 participants are placed in the relevant themes, as it is clearly seen in Table 3, there emerges a quite different situation from Table 2. Participants perceived very closely hedonic (53.5%) and eudaimonic well-being (47.5%) from culinary activities. Therefore, based on Stebbins’ classification, it can be said that culinary activities are leisure activities with both casual and serious characteristics.

**TABLE 3:** Hedonic/casual vs. eudaimonic/serious characteristics of cooking experience (*n* = 30).

Casual/hedonic characteristic (Σ_i =_ 46)	Σ_*i*_
**Pleasure (Σ_i =_ 23)**	
Happiness	14
Fun	8
Taking a stroll down memory lane	1
**Relaxation (Σ_*i* =_ 23)**	
Psychological relief/Staying strong	12
Getting away from bad thoughts	7
Calmness	4

**Eudaimonic/Serious Characteristic (Σ_i =_ 40)**	

**Knowledge, training and skills (Σ_i =_ 24)**	
Development of creativity	20
Learning to be patient	1
Learning to work more diligently	1
Increase of ability to observe	1
Development of health awareness	1
**Fulfilling and identity building (Σ_*i* =_ 16)**	
Belief in doing things better	12
Realizing self- sufficiency	4

*Since some participants stated more than one motivation factor, the total number of statements is above the total number of participants.*

When the hedonic well-being perception is examined, it is understood that “pleasure” and “relaxation” dimensions are repeated in a similar number of times. Many participants used expressions indicating both pleasure and relaxation in the same comment. Participants’ comments show that dealing with culinary activities in quarantine is a joyful activity and provides a great opportunity to produce something by having fun with family members with whom they could not spend time before. On the basis of Stebbins’ approach, the pleasure characteristic of culinary activities that provide “active entertainment” can be supported by the verbatim quotes from the different participants:

“The pandemic period is a boring time away from our school and when we cannot leave home. It was quite fun to spend this period in the kitchen. You don’t even understand how time passes” (Participant 6).

“It was emotionally pleasing to cook everything at home. But it is also a backbreaking work.” (Participant 12).

“I think cooking is a stress-relieving activity, and in this process, making dishes that I had previously consumed as ready-made but never tried to cook made me happy and encouraged” (Participant 20).

“At the end of a very entertaining and dynamic period, I felt the happiness of succeeding with the emergence of those beautiful products” (Participant 22).

“Trying new things and cooking my own food made me a little bit happy during these troubled times” (Participant 30).

Some of the participants stated that engaging in culinary activities helped overcome the psychological challenge of the lockdown. They also said that the time spent in the kitchen was flowing, making them forget the bad atmosphere a little bit and bringing peace. On the basis of Stebbins’ approach, the relaxation characteristic of culinary activities that provide “passive entertainment” can be supported by the verbatim quotes from the different participants:

“I can clearly say that you don’t understand how time passes. The kitchen makes people relax. Instead of thinking unnecessary things and feeling anxious, you feel relaxed” (Participant 1).

“I think being in the kitchen in such a period takes all my stress away, this process goes easier” (Participant 16).

“It really provided an emotional relief. I can say that it eliminates the bad psychological effects caused by constantly dealing with mobile phones and television” (Participant 3).

“Instead of distracting adverse situations, I feel peaceful as I bring out new products that will make me and the people around me happy. Also, it feels so good not to think of anything else” (Participant 11).

When the eudaimonic/serious characteristics perceived by the participants from their culinary activities were examined, it was understood that positive effects are perceived at the point of gaining special skills and knowledge (Knowledge, Training, and Skills) and self-actualization and self-enrichment (Table 3). These positive psychological effects also reveal the serious leisure feature of the kitchen activities, based on Stebbins’ classification. When the participants’ comments are analyzed in terms of their content, the most frequently expressed positive eudaimonic effect is the belief that the individuals’ creativity improved. Participants who did not spend such a long time in the kitchen before, stated that their knowledge and skills improved while applying what they learned from each other, from social media, or from sources such as TV programs. In addition, some of the participants stated that spending time in the kitchen made them more patient, taught them to work more diligently, and increased their observation skills and health awareness. These findings can be supported by the verbatim quotes from the different participants.

“I can clearly say that you don’t understand how time passes. The kitchen makes people relax. Instead of thinking unnecessary things and feeling anxious, you feel relaxed” (Participant 1).

“I think being in the kitchen in such a period takes all my stress away, this process goes easier” (Participant 16).

“It really provided an emotional relief. I can say that it eliminates the bad psychological effects caused by constantly dealing with mobile phones and television” (Participant 3).

“Instead of distracting adverse situations, I feel peaceful as I bring out new products that will make me and the people around me happy. Also, it feels so good not to think of anything else” (Participant 11).

“Being always at home encouraged me to try to cook novel foods. Seeing that I can do it has encouraged me even more. I’m constantly learning and trying to cook novel foods” (Participant 24).

“I started to observe better. I had the opportunity to focus more on things that I hadn’t noticed. In this way, I started to have a lot of ideas on how to produce more reliable food in a better, healthier and more hygienic environment.” (Participant 23).

“More experiences and tastes that increase with experience. Practicing, working fast and taking control of the kitchen. My ability to cook two or three meals at the same time has improved” (Participant 12).

“While cooking in the kitchen, depending on the situation, I learned to be fast, to wait, and to be patient” (Participant 5).

As mentioned above, other positive eudaimonic effects of cooking are about self-actualization and self-enrichment (Fulfilling and Identity Building). Individuals who spent more time in the kitchen during the quarantine period have refreshed their belief that they can do a job better whenever they want and improve their skills. In addition, it was observed that in a period when life is limited in many respects, individuals’ belief in self-sufficiency have increased thanks to culinary activities.

“I understood the belief of ‘I cannot cook that dish or dessert was a prejudice. It turns out that when I want to do something, I can do it just fine as long as I can spare time” (Participant 28).

“In this period, I understood more clearly that there is no limit to what I can do” (Participant 14).

“During the lockdown period, I needed things I never needed before (like baking bread). It made me feel safe to see that we could meet our own needs under any circumstances” (Participant 18).

“I noticed the fact that I was speeding up in my work when I focused or had enough time. Kitchen taught me this” (Participant 8).

## Discussion

The purpose of this study was mainly twofold; (i) explore the positive psychological impacts (hedonic/eudaimonic) of culinary activities during the COVID-19 lockdown days and (ii) to discover the leisure experience characteristics (casual/serious) of culinary activities. The participants’ culinary experiences were classified in terms of hedonic and eudaimonic well-being by combining with the Stebbins’s “Casual vs. Serious Leisure” theoretical framework. Thus, how participants perceived the time they spent in the kitchen during the pandemic lockdown period as a leisure recreation experience (casual vs. serious) and as a psychological well-being tool (hedonic vs. eudaimonic) was revealed. These perceptions were resolved as a result of the answers obtained from two open-ended questions. Based on qualitative analysis, positive psychological effects perceived by the participants were reported as (i) motivational well-being and (ii) experience well-being. In a simpler term, it was examined what the participants expected from being busy with culinary activities during the COVID-19 lockdown period and what they gained at the end of the period. Based on the answers, it was also revealed the leisure experience characteristic of culinary activities with reference to acquired psychological well-being.

The findings show that the well-being expectations (before) of people from culinary activities are quite different from the well-being outcomes (after) they get as a result of the time they spent in the kitchen. Participants’ answers showed that spending time in the kitchen in the first place was a casual experience and carried out with the aim of obtaining hedonic outcomes. The statements in the answers to the questions revealed that people were in a difficult psychological situation due to staying closed in at home during the COVID-19 outbreak. Therefore, the answers people gave to the question of “What were the expectations that pushed you to engage in culinary activities?” are gathered under the dimensions of “pleasure” and “sensory stimulation” referring to people’s endless pursuit of “happiness.” It has been observed that people, who started to spend time in the kitchen to be relieved from their boredom caused by staying closed in at home, turned to cook the dishes they have not tried to cook before due to the abundance of free time and perceive this activity as a quality time spent with their loved ones. In addition, the situational health and hygiene expectation arising from the pandemic also directed people to the kitchen. As a result, people perceived dealing with culinary activities during the quarantine period as a casual leisure activity expected to generate hedonic expectations. No answer showing eudaimonic well-being expectation was obtained from the answers given to the first question asked in the context of motivation.

The answers obtained from the question “What kind of benefits did spending time in the kitchen during the lockdown bring you?” It showed that there is a distinct difference between the participants’ expectations from spending time in the kitchen and what really happened at the end. While the participants basically had the aim of “happiness” (pleasure and relaxation) in their minds before dealing with culinary activities, it is obvious that they had long-lived positive psychological growths in addition to short-lived happiness. Although hedonic impacts are proportionally higher than eudaimonic effects, there is actually a remarkably close ratio to each other. In other words, the participants perceive the culinary activities during the COVID-19 quarantine as both casual and serious leisure activities, just as Stebbins (1997b) stated serious leisure experiences could produce hedonic beneficial outcomes as well. It is also clear that performing culinary activities produces more output in terms of overall psychological well-being compared to the motivation to start culinary activities. Culinary activities, which produced 51 codes in terms of motivational well-being expectation, produced 86 codes in the context of conclusive well-being effects. In this respect, it can be said that the culinary activities should be actualized in order to reveal the versatile effectiveness of these activities. In other words, intending to be in the kitchen and experiencing it in practice reveal differences in terms of psychological well-being outcomes.

When the answers to the second question are examined in terms of hedonic outputs, it is seen that the positive effects are concentrated in the feelings of happiness, psychological relief, fun, and steering away from bad thoughts, just as they are in motivational returns. In this sense, “positive effect” and “infrequent negative effect,” two of the three dimensions that Diener (1984, 1994) used in hedonic well-being measurement, emerged as a result of their activities, as well. When the answers were examined in terms of eudaimonic well-being outcomes, the two factors that emerged were named as “Knowledge, Training, and Skills” and “Fulfilling and Identity Building” based on the Stebbins’ serious leisure theoretical background (Stebbins, 1997b; Stebbins, 2008). Participants came out with eudaimonic well-being by gaining special skills and knowledge from the kitchen in which they entered with a hedonic motivation to experience new tastes. This, in the words of Stebbins (1997b), is extremely critical in understanding the casual and serious characteristics of an activity. Another eudaimonic perception of the participants who entered the kitchen with the motivation of “pleasure” and “relaxation” appeared at the “Fulfilling and Identity Building” point. The answers to this factor, which can be summarized as “self-actualization” and “self-enrichment” with Stebbins (2008) approach and as factors that “make one’s life fulfilling and worthwhile” in Ryff (1989) and Waterman et al.’s (2003) words, highlight the nature of culinary activities that provide more outcomes than “pure happiness.” It is understood that during the COVID-19 quarantine process, which is a psychologically challenging period, people have learned to draw wider inferences about life by realizing their own potential thanks to culinary activities.

Leisure cooking activities discussed considering that they will contribute positively to the psychological well-being of people during the COVID-19 lockdown period are of course not a new topic for the literature. Cooking activities have been discussed in the literatures of psychology, public health, and leisure with the aim of examining their positive effects on the psychological, psychosocial and physical well-being. Systematic review of Farmer et al. (2018) reveals the fact that most of the research focused on the psychological, psychosocial and physical benefits of cooking activities on disabled, sick, elderly, and disadvantaged individuals (Tarasuk and Reynolds, 1999; Marquis et al., 2001; Fitzsimmons and Buettner, 2003; Hill et al., 2007; Jyväkorpi et al., 2014; Barak-Nahum et al., 2016) and social benefits of community based cooking programs on public (Engler-Stringer and Berenbaum, 2007; Lee J. H. et al., 2010; Herbert et al., 2014). All these studies had a use of cooking and intervention settings. The findings of those studies revealed that both inpatient and community-based cooking interventions yielded positive influences on improvement of socialization (Tarasuk and Reynolds, 1999; Marquis et al., 2001; Engler-Stringer and Berenbaum, 2007; Lee J. H. et al., 2010), a decrease in negative feelings (e.g., Fitzsimmons and Buettner, 2003; Hill et al., 2007; Barak-Nahum et al., 2016) and an increase in psychological well-being (e.g., Jyväkorpi et al., 2014; Barak-Nahum et al., 2016). These priceless findings highlight the hedonic well-being impact of cooking activities in different samples through a variety of intervention settings.

Although the psychological atmosphere experienced by the individuals in the sample of this study is different than others, it should not be forgotten that the COVID-19 pandemic has been a disease that affects all segments of the society. In particular, the unpredictability of the negative impacts of the virus created great anxiety and fear in all. Therefore, it is a particularly important result that the effects of cooking interventions in patients, elderly or disadvantaged individuals in the above research are similar to the hedonic characteristics of the effects of this study, which was carried out without any intervention. Reicks et al. (2018), who analyzed studies that investigated the topic of “impact of cooking and home food preparation” again through interventions, showed that among 34 studies, seven studies conducted with samples of healthy (Condrasky et al., 2013; Flego et al., 2014; Garcia et al., 2014), disadvantaged (May et al., 2014), and diabetic (Dasgupta et al., 2012; Hossain et al., 2015) adults had significant gains in cooking skills and knowledge. From this point of view, it is understood that cooking activities carried out through interventions, although it is limited to “Knowledge, Training and Skills” reveal eudaimonic effects as well. Even the eudaimonic outcomes revealed by cooking activities in this study is similar to those realized through interventions in the literature, this study promotes a useful contribution to the literature through the “Fulfilling and Identity Building” characteristics of eudaimonic well-being. As aforementioned, there are limited number of studies on the psychological effects of cooking without any intervention in the pre-COVID-19 period (Daniel et al., 2011; Mosko and Delach, 2020) and during the COVID-19 pandemic period (Easterbrook-Smith, 2020; Gammon and Ramshaw, 2020). When the findings of this study are compared with those four valuable research’s results, it seemed that emerged hedonic and eudaimonic outcomes are similar, although more comprehensive outcomes emerge in this study. On the other hand, aforementioned four studies did not take the culinary activities in terms of well-being classifications and leisure experience characteristics.

## Conclusion, Limitations, and Future Research

Consequently, it is possible to say that culinary activities were considered as an important “escape” activity during the COVID-19 period, especially for people who suddenly faced a lockdown almost no one is accustomed to. Individuals who entered the kitchen with expectations of pure happiness and relaxation, left from the kitchen by getting informed, improved skills and knowledge and identity building outcomes in addition to pleasure and relaxation. Therefore, it could be asserted that culinary activities outweigh even people’s expectations from themselves. Consequently, these findings reveal an apparent view on the hedonic and eudaimonic nature of culinary experiences during the COVID-19 lockdown period. Stebbins (2004) suggested that the most optimum expectation from a leisure activity is the ability to present hedonic and eudaimonic outcomes together. Hence, it is possible to say that culinary activities provide a suitable leisure experience to meet these expectations. On the other hand, the emerged findings that culinary activities have not just casual but also serious experience characteristics, blur the common belief regarding serious leisure experience could be produced through *“competitive or non-competitive sport activities and highly social activities”* (Voigt et al., 2010). Prominently, the interviewees’ responses addressing the theme of “Fulfilling and Identity Building” highlight the serious experience characteristics of culinary experience. Therefore, well-being impacts of culinary activities could be discussed more comprehensively in cooperation with leisure, recreation and psychology researchers. For example, this study showed the serious/eudaimonic experience characteristics of the culinary activities during the COVID-19 lockdown period with two themes: “Knowledge, Training, and Skills” and “Fulfilling and Identity Building.” However, serious/eudaimonic experience characteristics of a leisure involves; “Belongingness to Special Social World,” “Career Development,” “Effort and Perseverance as well.” Through the additional studies these factors could be explored or new factors that shape serious characteristics of culinary activities could be discovered.

As of December 2020, when this article was written, a variety of clinical vaccine development processes to end the coronavirus pandemic are ongoing all over the world. Although there are great efforts to implement these vaccines in the first quarter of 2021 as the earliest date, it is clear that the issue of efficiency and distribution of the vaccines remain as major problems. In addition, while new records are broken in terms of both the number of cases and deaths all over the world every day, new lockdown, closing and social distancing measures are being taken in many countries throughout the winter months. Therefore, it seems that culinary activities will maintain its importance as a way for people to keep their psychology strong in their struggle with this difficult and invisible enemy for a while. At this point, a suggestion can be presented to the practitioners. Research results show that culinary activities can provide people with outcomes that are entertaining, informative and additionally that will enable them to know themselves better and realize its’ true potential. Therefore, in this period that the negative effects of COVID-19 still affect the social life of the all human-beings all over the world, both private and non-profit institutions and organizations should carefully emphasize the hedonic and eudaimonic outputs of culinary activities in their marketing communications while trying to attract people to cooking classes whether online or face to face. On the other hand, culinary activities can be recommended by psychologists as a versatile remedy in order to reduce or eliminate psychological traumas experienced in almost all demographic segments of society.

Like many studies, this study has number of important limitations. The first limitation of the study is the process of data collection and procedure. Due to the circumstances of the COVID-19 lockdown, the method used in the study is “qualitative online survey” instead of the traditional method based on collecting data face to face and voice recording. Although this choice was an obligation for this study, it is necessary to say that semi-structured or unstructured face to face interviews is the most accurate method for the most comprehensive inferences in accordance with the nature of qualitative research. Another limitation of the research arises from the sampling technique. To reach the maximum variation in the sample, heterogeneous purposive sampling method was used. This let the researchers take advantage of referring to convenience and snowball samplings, as well. As a result, the minimum age was 20 while the oldest was 53. However, the people under the age of 20 and over the age of 60 are the most significantly affected ones by quarantine measures. Additional studies could add these groups into the samples. Another limitation arises from the analysis method used in the study. The five-stage content analysis, independent from a statistical software package, was carried out using Microsoft Office programs “Word” and “Excel.” Analyzes can be made with different qualitative statistical analysis programs in future research.

In parallel with the aim of the study, solely people who involved in culinary activities during the lockdown days were included into the study. Nevertheless, it would have been great to use a control group that does not engage in cooking during the pandemic to better demonstrate how cooking helps individuals more learn about themselves. Future researchers could fill this gap with experimental designs. The findings obtained in the study were not classified and interpreted according to categorical characteristics such as certain age groups, gender, marital status, and medical record. Although the psychological impact of COVID-19 on people concerns every segment of the society, from children to the elders, it will be useful to understand whether the culinary experiences that offer a positive psychological effect show significant differences according to demographic characteristics. Besides, in this study the data collected were from the people who live in Turkey. Further studies could collect data from different countries and compare the findings to reveal how the positive psychological well-being outputs and Stebbins’s serious leisure experience framework are perceived differently. This study focused on positive impacts of unique activity (cooking) on human well-being. This helped individuals cope with psychological distress during the lockdown due to COVID-19. Future research could explore the negative impacts of cooking on psychological well-being, notably from the viewpoint of whom is the responsible from cooking at home. Finally, since this study designed with qualitative methods, statistical relationships between variables were not considered. If it is intended to investigate the effects of perceived hedonic and eudemonic well-being from in-door culinary activities on variables such as life quality and life satisfaction, quantitative research methods can be used.

## Data Availability Statement

The raw data supporting the conclusions of this article will be made available by the authors, without undue reservation.

## Ethics Statement

The studies involving human participants were reviewed and approved by Mersin University, Social Sciences Ethics Committee. The patients/participants provided their written informed consent to participate in this study.

## Author Contributions

OG and MH co-designed the study. OG and MH collected the data together, while OG analyzed the data and reported the findings. Sections of discussion and conclusion were written collectively. At the end, all sections of the manuscript checked collectively.

## Conflict of Interest

The authors declare that the research was conducted in the absence of any commercial or financial relationships that could be construed as a potential conflict of interest.
